# Cost and Cost-Effectiveness of Smear-Positive Tuberculosis Treatment by Health Extension Workers in Southern Ethiopia: A Community Randomized Trial

**DOI:** 10.1371/journal.pone.0009158

**Published:** 2010-02-17

**Authors:** Daniel G. Datiko, Bernt Lindtjørn

**Affiliations:** 1 Centre for International Health, University of Bergen, Overlege Danielsens Hus, Bergen, Norway; 2 Southern Nations, Nationalities and Peoples' Regional State Health Bureau, Hawassa, Ethiopia; London School of Hygiene and Tropical Medicine, Peru

## Abstract

by HEWs in the health posts and general health workers at health facility were compared along a community-randomized trial. Costs were analysed from societal perspective in 2007 in US $ using standard methods. We prospectively enrolled smear positive patients, and calculated cost-effectiveness as the cost per patient successfully treated. The total cost for each successfully treated smear-positive patient was higher in health facility ($158.9) compared with community ($61.7). Community-based treatment reduced the total, patient and caregiver cost by 61.2%, 68.1% and 79.8%, respectively. Involving HEWs added a total cost of $8.80 (14.3% of total cost) on health service per patient treated in the community.

**Conclusions/Significance:**

Community-based treatment by HEWs costs only 39% of what treatment by general health workers costs for similar outcomes. Involving HEWs in TB treatment is a cost effective treatment alternative to the health service, to the patients and the family. There is an economic and public health reason to consider involving HEWs in TB treatment in Ethiopia. However, community-based treatment requires initial investment to start its implementation, training and supervision.

**Trial Registration:**

ClinicalTrials.gov NCT00803322

## Introduction

Ethiopia has one of the highest tuberculosis (TB) burdens in the world [1]. Directly observed treatment short course (DOTS), the World Health Organization (WHO) recommended TB control strategy, was started in 1995 in Ethiopia by the National TB and Leprosy Control Programme (NTLCP), being decentralized to hospitals and health centres [2]. However, less than half of the population has access to the health service [3]. Thus, many TB patients remain undiagnosed, untreated and continue transmitting the infection. The interaction between TB and human immunodeficiency virus (HIV) infection has fuelled the TB burden and affected the already overstretched health service, which needs alternative ways of making the service accessible [4].

Under the NTLCP, there are three levels of function for coordinating TB control in hospitals and health centres: regions, zones and districts. TB diagnosis and treatment is provided by general health workers (GHWs) in health facilities [5]. The treatment success rate (TSR) of smear-positive cases in the study area was 76% (unpublished three year review of TB programme performance in the study area), while 84% at the National level. The case detection rate (CDR) of smear-positive cases was 41% in the study area and 27% at the National level, far below the target of 70%. However, the cost implication for the health service and the community has not been estimated.

In 2004, the Government of Ethiopia launched a community-based initiative focused on disease prevention and health promotion to ensure equitable access to health service. To this end, health extension workers (HEWs) were trained and deployed to each kebele (the lowest administrative unit in Ethiopia) to provide health service. The HEWs function from an operational unit - a health post in each kebele, receiving training on TB as part of communicable disease prevention and control [6]. However, community DOTS was not yet implemented and HEWs were not providing directly observed treatment (DOT) to TB patients [5].

Studies show that involving community health workers in TB control is cost-effective in improving the treatment success compared with health facility-based DOTS [7], [8], [9], [10], [11], [12], [13], [14], [15]. Community DOTS requires supervision, some initial investment and a well-coordinated TB control programme. Its effectiveness therefore depends on how well the health system functions in coordination with the community [16].

We conducted a community randomized trial (CRT) in Southern Ethiopia to determine whether involving HEWs in TB control would improve smear-positive CDR and TSR compared with health facility-based TB treatment. We found both improved CDR (122% *vs.* 69%, p<0·001) and TSR (89% *vs* 83%, p = 0·012) in the community-based DOT (CDOT) compared with the health facility-based DOT (HFDOT) [17]. Therefore, determining whether involving a community-based approach is also more cost-effective would seem a relevant issue for policy- and decision making. To our knowledge, there have been no studies of cost and cost-effectiveness of alternative ways of treating TB in Ethiopia. In this study, we aimed to determine the cost and cost-effectiveness of involving HEWs in TB treatment in Southern Ethiopia. This paper presents an ancillary cost-effectiveness analysis of data from a RCT, from which the main outcomes have already been published.

## Methods

The protocol for this trial and supporting CONSORT checklists are available as supporting information; see Checklist S1, Flowchart S1 and Protocol S1. Full description of trial methodology is given in the paper reporting main trial findings [17]. Briefly, two treatment options of treating smear-positive patients were compared: health facility and community DOT (CDOT - the intervention).

### Health Facility-Based DOT (HFDOT)

TB patients receive treatment under the direct observation of GHWs in hospitals and health centres. They visit health facilities daily for two months during the intensive phase. During the continuation phase, patients visit health facilities once a month to collect drugs but take the drugs unsupervised.

### Community DOT (CDOT): The Intervention

TB patients visit the health post daily for two months during the intensive phase to receive treatment under the direct observation of HEWs in their kebele. During the continuation phase, patients collect drugs from the HEWs on a monthly basis.

Trained HEWs and GHWs prospectively collected the cost data by using a structured questionnaire. GHWs also used a checklist to observe the conduct of DOT in the health facilities and the kebeles.

### Costing

Costs were assessed from a societal perspective in 2007 in US dollars, using recommended standard methods [18]. We classified costs in to programme and patient costs. Direct cost refers to patient's out-of-pocket expenses for seeking treatment, while indirect cost refers to the cost of the time spent by the patient or their caregivers or freed by the programme. Weighted mean cost was calculated to costs related to patients and caregivers. In this study, hereafter, cost values refer to mean cost values per successfully treated smear-positive TB patient.

#### Programme costs

Programme costs are the health service costs including the expenses required to establish the health service, and run the TB programme in the districts and health facilities including the health posts in the kebeles. The average cost for each component of treatment (drugs, sputum examination, treatment and other medical expenses) was calculated from the quantity and unit prices of resources. Time costs were estimated from the health facility providing DOT to the patient's place of residence. Joint costs (cost items shared by two or more services) were allocated to TB patients based on the proportion of the total health facility visits which they accounted for and the associated health workers time. Annuitization was done on the basis of the expected useful life of 30 years for buildings, 10 years for cars and equipment and 5 years for motorcycles [19]. The base year for valuing costs was 2007, and the exchange rate was 8.6 Ethiopian Birr to US $1.

The cost of HEWs, part of the health service, included the time spent for treatment supervision in the kebele, travel time and expenditures associated with visits to the health facilities to collect drugs. The time costs were converted to a monetary value based on the monthly income of HEWs in US dollars. The cost of training and supervision was also included.

#### Patient costs

Patient costs include the costs related to the TB patient and their caregiver. The costs were estimated for the smear-positive patients and their caregivers using a structured questionnaire. TB patients and the caregivers were asked about the travel time and expenses associated with visits to HEWs in the health post to take drugs. This included transport, food and other costs. The cost data was collected for all caregivers who accompanied the patients to health centres and health posts. Travel time was estimated from the patient's home to the health post in the kebele. The time costs were converted to a monetary value based on unskilled wage rates [18] which was US$1.39 per day (US$0.17 per hour) in the study area.

The cost data was case specific for all study participants and was standard in each arm of the intervention. At least ten visits in the intensive phase and six visits in the continuation phase were used as a standard for smear-positive patients and care givers for both the CDOT and HFDOT.

For each treatment option, average costs were multiplied by the number of times each cost was incurred to calculate the cost per patient successfully treated. For each kebele, we calculated summary values of costs and then used an independent sample *t* test, weighted by cluster size, to compare the mean costs using kebele as a unit of analysis.

The data sources were budget and expense files of the districts finance and health offices, health facilities, health workers' payroll, drug and supply prices, funds used from research projects (training, supervision and review of activities), TB control programme, bank reports and interview of the study participants.

### Effectiveness

The measure of effectiveness was based on sputum smear results at the end of the 2^nd^, 5^th,^ and 7^th^ months of treatment. Patients with at least two negative smears including the smear at the 7^th^ month were reported as cured. Patients who finished the treatment but did not have the 7^th^ month smear result were reported as treatment completed. We used TSR as a measure of effectiveness, which is a standard indicator used by WHO to measure programme success and which has been adopted by the NTLCP in Ethiopia. TSR was calculated as the sum of the number of TB patients who were cured and the number of TB patients for which treatment was completed divided by the total number of smear-positive cases reported, expressed as a percentage [1], [5]. The effectiveness data was obtained from CRT. Briefly, we calculated the summary values of TSRs for each kebele. Then we used an independent sample *t* test, weighted by cluster size, to compare the mean TSR using the kebele as a unit of analysis. This is robust for cluster level analysis of binary outcomes [20].

In a no intervention scenario (“do-nothing alternative”), a self-cure rate of 20% was used but 0% for HIV infected TB patients. The reported rate of TB-HIV co-infection in southern Ethiopia was 17.5% [21]. The self-cure rate was calculated using the following formula: [(estimated percentage of HIV+ patients x 0) + (estimated percentage of TB patients who are not HIV infected x 20)]/100 [22].

### Cost-Effectiveness

Cost-effectiveness was calculated as the average cost per patient treated successfully. This was done by dividing the total cost by the number of TB patients successfully treated for each of the two treatment options, the CDOT and HFDOT.

### Sensitivity Analysis

Sensitivity analysis determines the level of uncertainty in the components of the evaluation by repeating the comparison between cost items and consequences while varying the assumptions underlying the estimates. A one-way sensitivity analysis varies one cost item at a time while others are held at base value to measure its impact on the results of the evaluation [18], [23]. We performed one-way sensitivity analysis to assess the robustness of the results to changes in the cost values. We varied one cost variable at a time, repeating the analysis for the cost items. We based the uncertainty analyses on the minimum and maximum values of mean travel time, transport and total cost in our study. We used the 95% confidence interval of the effectiveness for the treatment outcome.

### Ethical Clearance

The Ethical Review Committee of Southern Nations, Nationalities and Peoples' Regional Health Bureau approved the study. We first discussed the aim of the study with the TB programme managers and kebele leaders about community- based TB care and obtained permission to proceed. Then we explained the aim of the study to the study participants and enrolled them after obtaining informed consent. The study participants were also informed about the right to refuse or withdraw from the study. The Ethical Review Committee approved verbal consent, in adherence to NTLCP recommendations.

## Results

Two hundred and twenty-nine smear-positive patients were enrolled. We interviewed 161smear-positive patients and 113 care givers in the CDOT and 68 smear-positive patients and 97 caregivers in HFDOT. More women were enrolled in CDOT 62% (99 of 161 patients) than HFDOT 37% (25/68). Regarding literacy, 63% of the patients (93/148) from community and 55% (33/60) from facility were literate. Regarding marital status, 63% patients (93/148) from community and 71% (47/66) from facility were married (Table 1).

**Table 1:** Baseline characteristics of smear-positive tuberculosis patients in Southern Ethiopia.

Variable	Community DOT	Health facility DOT
**Mean age (SD)**	26.8 (13.7)	25.2 (11.8)
**Gender**		
Men	62 (38.5%)	43 (63.2%)
Women	99 (61.5%)	25 (36.8%)
**Education**		
Illiterate	55 (37.2%)	27 (45.0%)
Literate	93 (62.8%)	33 (55.0%)
Missing	13	8
**Occupation**		
Student	38 (32.2%)	16 (26.7%)
Farmer	34 (28.8%)	24 (40.0%)
Housewife	38 (32.2%)	18 (30.0%)
Others	8 (6.8%)	2 (3.3%)
Missing	42	8
**Marital status**		
Single	51 (34.5%)	19 (28.8%)
Married	93 (62.8%)	47 (71.2%)
Widowed/divorced	4 (2.7%)	0 (0.0%)
Treatment outcome*		
Cured	132 (82.0%)	56 (82.4%)
Treatment completed	29 (18.0%)	12 (17.6%)

*A smear-positive tuberculosis patient with at least two negative smears including that at 7^th^ month was reported as cured, while a patient who finished the treatment but did not have the 7^th^ month smear result was reported as treatment completed.

### Costs

#### Programme costs: the health service and health extension workers costs

The health service invested US$73.5 for HFDOT and US$7.9 for CDOT. The cost of anti-TB drugs for a patient was US$22.1. The cost of training was US$10.0 in HFDOT and US$5.1in CDOT. Similarly, the cost of supervision was US$10.9 in HFDOT and US$5.9 in CDOT.

The travel time (estimated travel cost) for HEWs was 19.7 hours (US$5.1). The transport and food costs were US$0.9 and US$2.8, respectively. Therefore, the HEWs total cost per patient was US$8.8, accounting for 14.3% of the total cost per patient for CDOT.

#### Patient costs: the patient and caregiver costs

**Figure 1:**
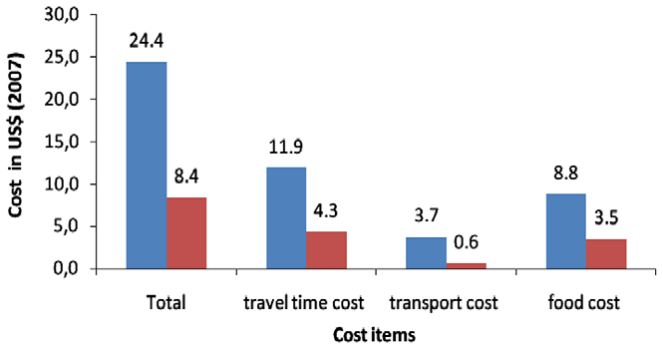
Tuberculosis patient costs under DOT options. Blue bar - Health facility DOT. Red bar - Community DOT.

The patient costs are described as follows. The mean and standard deviation (SD) of travel time (estimated travel cost) was 27.6 hours (US$4.3, SD = 1.9) in CDOT and 68.9 hours (US$11.9, SD = 5.2) in HFDOT (p<0.05). The transport cost was US$0.6 (SD = 1.2) in CDOT and US$3.7 (SD = 10.5) in HFDOT (p = 0.013). Similarly, the associated food cost was (US$3.5, SD = 2.9) in CDOT and US$8.8 (SD = 5.2) in HFDOT. The direct patient cost was lower in CDOT (US$4.1, SD = 3.0) than HFDOT (US$12.1, SD = 10.7) (p<0.05). The total patient cost was lower in CDOT (US$8.4, SD = 3.9) than HFDOT (US$24.4, SD = 12.2) (p<0.05). The total cost in CDOT was lower than HF DOT by 63.9% (Figure 1).

**Figure 2:**
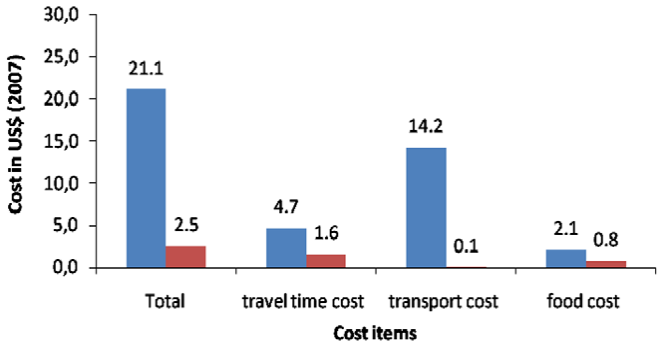
Caregiver costs under DOT options. Blue bar - Health facility DOT. Red bar - Community DOT.

The caregiver costs are described as follows. The travel time (estimated travel cost) was 9.9 hours (US$1.6, SD = 1.5) in CDOT and 16.5 hours (US$4.7, SD = 5.7) in HFDOT (p<0.05). The transport cost was lower in CDOT (US$0.1, SD = 0.9) than HFDOT (US$14.2, SD = 43.6) (p = 0.006). Similarly the associated food cost was lower in CDOT (US$0.8, SD = 1.2) than HFDOT (US$2.1, SD = 2.8) (p<0.05). The total caregiver cost was lower in CDOT (US$2.5, SD = 2.7) than HFDOT (US$21.1, SD = 50.6) (p = 0.002). The total care giver cost in CDOT was lower than HF DOT by 88.2% (Figure 2).

**Figure 3:**
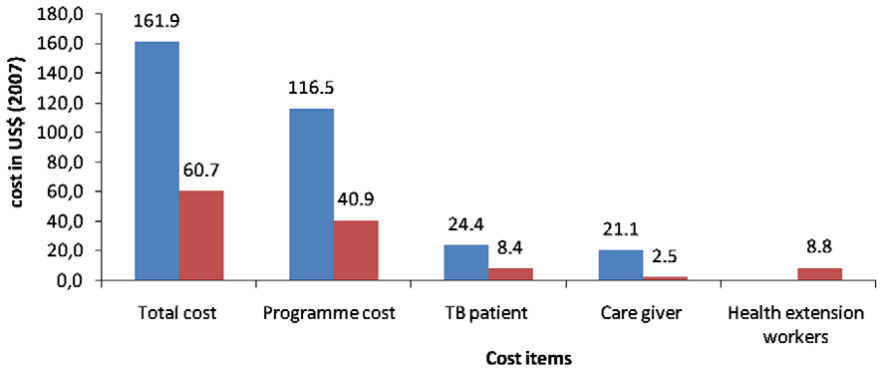
Costs per successfully treated smear-positive tuberculosis patient. Blue bar - Health facility DOT. Red bar - Community DOT.

The total cost (patient and programme cost) per successfully treated smear-positive patient was higher in HFDOT (US$161.9) compared to CDOT (US$60.7). The total cost in CDOT was lower than HF DOT by 62.6% (Table 2 and figure 3).

**Table 2:** Average cost per patient for treatment options of smear-positive tuberculosis patients in Southern Ethiopia 2006/07.

Cost items	Community DOT		Health facility DOT	
	quantity	mean unit price in US$	quantity	mean unit price in US$
**Programme costs**				
Running TB programme	1	7.9	1	73.5
Training and review meeting	1	5.1	1	10.0
Drugs and supplies	1	22.1	1	22.1
Supervision	8	5.9	3	10.9
*Health extension workers cost*				
Direct cost of visit	8	3.7		
Indirect cost	8	5.1		
*Total programme cost*		*49.8*		*116.5*
**Patient costs**				
**TB patient**				
Direct cost of visits*	66	4.1	66	12.5
Indirect cost		4.3		11.9
**Caregiver**				
Direct cost		0.9		16.3
Indirect cost		1.6		4.7
*Total patient costs*		*10.9*		*45.4*
**Total costs**		60.7		161.9

* Patients visited the health facilities in health facility DOT and health posts in community DOT. Health extension workers visited the health facilities monthly to collect drugs. Direct cost implies out-of- pocket expenses and indirect cost implies travel time cost.

### Effectiveness

In the CRT, smear-positive patients received DOT, 230 under HEWs in the community and 88 under GHWs in health facilities. In the community-based approach, of the 230 patients, 172 (74·8%) were cured and 33 (14·3%) completed treatment. Of the 88 patients treated in the health facilities, 60 (68·2%) were cured and 14 (15·9%) completed treatment. The mean TSR was higher in CDOT (89.3%) than HFDOT (83·1%). The mean and its difference being 6.2% (1.4% - 10.9%, p = 0·012). The details are given elsewhere [17]. Based on the reported 17.5% TB-HIV co-infection rate in smear-positive patients in Southern Ethiopia [21] and using the formula to calculate self-cure, cure without treatment (given above in the methods section under ‘effectiveness’), we found TSR of 80.8% for HFDOT and 86.9% for CDOT.

### Cost-Effectiveness

The cost per successfully treated patient was US$161.9 and US$60.7 in HFDOT and CDOT, respectively. CDOT reduced the total cost per successfully treated patient by 62.6% (Table 2 and figure 3). Based on the cost and effect estimates of no intervention (US$0, 17%), HFDOT (US$161.9, 83.1%) and CDOT (US$60.7, 89.3%), the incremental cost-effectiveness ratio of running HFDOT and CDOT from a do-nothing alternative was 2.4 and 0.8, respectively. The incremental cost-effectiveness ratio of HFDOT to CDOT was -16.3.

### Sensitivity Analysis

The sensitivity analysis showed CDOT to be a more effective and less costly approach compared to HFDOT on varying estimates of the main cost items (Table 3).

**Table 3:** Variation in cost-effectiveness ratio upon changes in input variables.

*Input variables*	*Input values*		CER* CDOT		*Input values*		*CER HFDOT*	
	Min.	Max.	Min.	Max.	Min.	Max.	Min.	Max.
TB patient time cost	0.51	11.16	51.12	55.95	3.16	27.21	125.29	150.33
Care giver time cost	0	7.17	54.08	59.19	0	18.95	133.87	160.63
HEW† time cost	3.63	8.94	55.04	60.24				
TB patient cost	0.65	17.37	55.06	60.26	12.32	94.88	127.52	153.01
Caregiver cost	0	13.19	55.43	60.67	0	177.27	131.56	157.86
HEW cost	3.63	30.05	55.62	60.88				
Discount rate	0	3%	59.83	61.68	0	3%	154.21	158.98
TSR-N‡	82.22%	91.74%	77.94	86.97				
TSR-N					77.79%	89.85%	207.50	259.71
TSR§	85.11	93.15	55.74	61.00				
TSR					76.45	91.73	136.27	163.51

* Cost-effectiveness ratio.

† Health extension worker.

‡ Treatment success rate excluding self-cure.

§ Treatment success rate.

## Discussion

### Interpretations and Overall Evidence

In Ethiopia, DOTS coverage was reported to be 100% (implemented in all hospitals and health centres). However, case finding and TSRs are below the WHO targets [1]. In such a setting, it is relevant to ask how this could be improved. Improvement may be achieved by more efficient intervention for identifying TB cases, including providing treatment at a lower cost [24]. In our study, the cost of treating a patient in health facilities was 2.7 times higher than the cost of treating a patient in the community-based approach inclusive of the initial investment for implementation, training and supervision of CDOT. CDOT improved the TSR by 6.2% and reduced the cost of treating a patient by 62.6%. This shows that more patients could be successfully treated with the same amount of resources by using CDOT instead of HFDOT.

The main reason for the reduction in cost of the community-based approach was the reduction in the travel distance and related costs as the patients visited the HEWs in the health post, which was located nearer to where the patients lived. The reduction in caregiver and patient costs results in a slight increase over the health service cost. However, from a societal perspective, the gain in terms of cost and health benefits is huge. Thus, involving HEWs in TB treatment is an attractive economic option to the health service and to the patients and their caregivers.

Decentralization of the DOTS programme improves the TSR [25], [26]. A community-based approach is found to be more effective and cost-effective as it overcomes the limitation of reliance on health facilities in providing access to TB care [27], [28], [29]. It also consistently reduces the cost of treatment even in a decentralized health service [16]. In our study, the cost per successfully treated patient was low (US$61) compared with studies from Malawi (US$201) and Botswana (US$1657). Similarly, the reduction in average cost per patient treated in our study was 63% compared to those reported for South Africa (36%) and Kenya (65%) [11], [12], [13], [14]. The main reason could be that Ethiopia is a low-cost country with low salaries. Also, we did our study in a rural setting as opposed to an urban setting.

The gain in effectiveness of the CDOT was mainly due to the reduced travel distance that reduced the cost, and time lost on travel to receive treatment. In settings with low health service coverage the significance of CDOT is high. CDOT could complement the existing health service to improve the access and success rate TB programmes in countries like Ethiopia where CDOT has not yet been implemented on a national scale.

The strength of the study is that the data was prospectively collected in CRT. We adhered to the routine care for treatment and outcome measures as recommended by the NTLCP, which did not require extra visits by the patients because of the community-based approach. We used HEWs living in the kebeles, who were employed to provide a health service that favoured the sustainability of the community approach in the Ethiopian health system as opposed to other community approaches whereby the community health workers have been used for only short periods. We conducted our intervention under approved programme conditions and prospectively collected cost data that reduced the chance of recall bias. We included all cost categories in the sensitivity analysis that reduced the chance of selection bias. Moreover, the long period of the observation (September 2006 to April 2008, i.e., 20 months) may have contributed to the consistency of the data [18]. In our study we did not have drop outs of HEWs due to the fact that HEWs were selected from the kebeles they live in. However, in the future, the possibility of training new HEWs and providing refresher training to the already trained HEWs should be considered. This also applies to the general health workers involved in TB control in health centres and hospitals that have higher drop outs. Therefore, the estimated cost required for CDOT will still remain lower than HFDOT for similar outcomes.

The government of Ethiopia has already increased the uptake and training of HEWs in the country to ensure and deploy two HEWS per kebele. Thus, doubling HEWs per kebele has already started at the end of the first year of the intervention in 2007 before drop outs occur at least in our study area. Therefore, it only requires training HEWs which was two days in case of our study to enrol them in community based TB control activities to achieve the outcomes reported in our intervention.

A major limitation of the study was that we based our estimation on the time converted into monetary value for which there is no agreement among experts [18]. It was also difficult to get reliable income data for rural areas. Therefore, we based our estimate on the wage of unskilled labour. This might have underestimated the time cost. In treatment outcomes like deaths and defaulters the cost was not captured for the total follow up period due to the nature of the outcome. In such cases, the distance from the health institutions and the related high cost could be the plausible explanations for such outcomes mainly in HFDOT. Therefore, the cost of treating smear-positive patient could be on the lower side, an underestimate, in both arms but mainly in HFDOT where distance and related cost was high. Using one-way sensitivity analysis, where we varied one cost item at a time, might not have captured the interaction between cost items. The economic and public health benefit of treating TB patients in terms of disease transmission, averting death or increasing productivity was not the scope of the study.

### Generalizability

Our study area was a densely populated agrarian community in Ethiopia. This area is typical of the rural population of Ethiopia, representing 85% of the total population where, high treatment success rates are not achieved because of the limited health service coverage and shortage of health workers. With health posts in each kebele and the huge number of HEWs, more cost-effective approaches are needed. As opposed to the study period where there was only one HEW per kebele, now two HEWs are deployed to rural kebeles in Ethiopia. Thus, we believe that our findings are applicable in similar settings. For example our approach could be adopted in other regions or countries where two HEWs work in each rural kebele. In addition, the Federal Ministry of Health of Ethiopia has assigned full-time public health nurses as supervisors of HEWs that favour implementation of CDOT. We presented the results of the study at a NTLCP review meeting.

In conclusion, community DOT costs only 37% of what HFDOT costs for similar outcomes. For the same amount of money in health facilities, at least two smear-positive patients could be treated under a community-based approach. There are both economic and public health reasons to consider involving HEWs in TB treatment by the NTLCP of Ethiopia. However, due attention should be paid to ensuring initial start up investment to implement CDOT, training and supervision.

## Supporting Information

Checklist S1 (0.06 MB DOC)

Flowchart S1 (0.03 MB DOC)

Protocol S1 (0.44 MB DOC)
