# Supplementary material for: Cost and Cost-Effectiveness of Smear-Positive Tuberculosis Treatment by Health Extension Workers in Southern Ethiopia: A Community Randomized Trial
Source: PLoS One. 2010 Feb 17;5(2):e9158. doi: 10.1371/journal.pone.0009158 (PMC2822844; doi:10.1371/journal.pone.0009158)
Supplement: Flowchart S1 — (0.03 MB DOC) [file pone.0009158.s002.doc]

Enrolment

allocation

Follow up

Analysis

Assessed for eligibility

51 communities

Excluded (n = 0)

Randomized

51 communities

Allocated to intervention

30 communities

161 tuberculosis patients

Allocated to control

21 communities

68 tuberculosis patients

Lost to follow up

7 tuberculosis patients

Allocated to intervention

3 tuberculosis patients

Analyzed

68 tuberculosis patients

Analyzed

161 tuberculosis patients
