# Supplementary material for: Cost and Cost-Effectiveness of Smear-Positive Tuberculosis Treatment by Health Extension Workers in Southern Ethiopia: A Community Randomized Trial
Source: PLoS One. 2010 Feb 17;5(2):e9158. doi: 10.1371/journal.pone.0009158 (PMC2822844; doi:10.1371/journal.pone.0009158)
Supplement: Protocol S1 — (0.44 MB DOC) [file pone.0009158.s003.doc]

**IMPROVING COMMUNITY based TUBERCULOSIS care IN SOUTHERN ETHIOPIA**

**PI**

**Daniel G. Datiko,**

**PhD candidate**

**Supervisor**

**Prof. Bernt Lindtjørn,**

**Centre for International health**

**Bergen University, Norway**

**Summary**

**Improving community based tuberculosis care in southern Ethiopia**

Ethiopia ranks 7^th^ among high tuberculosis burden countries in the world. After implementing DOTS for more than ten years, the case detection rate of smear positive cases is only 36 %, far below global target. Therefore, tuberculosis control programme should find alternative ways to complement its activities.

The general objective of the study is to improve interventions to carry out community-based tuberculosis care in southern Ethiopia. The specific objectives are:

1. To evaluate the diagnostic value and applicability of community based TB screening tool,

2. To estimate the treatment outcome of TB patients supervised by community health workers

3. To find out the costs per tuberculosis patient treated successfully

4. To find out the acceptability of community-based directly observed short course therapy

5. To find out the proportion of tuberculosis patients infected human immunodeficiency virus

This is a randomized controlled community trial to estimate the treatment outcome of tuberculosis patients treated by community based directly observed short course therapy. The investigator will select matched clusters and randomly allocate to intervention and non-intervention clusters. Tuberculosis patients in the intervention clusters will start supervised short course therapy in their community. The cost per patient treated will be determined with the intervention. In addition, cross-sectional studies will be conducted to find out the acceptability, TB HIV co-infection and evaluate of diagnostic tool for community-based tuberculosis care.

The investigator will get ethical clearance from University of Bergen and Regional Health Bureau in southern Ethiopia. In collaboration with programme coordinators, the investigator will train health workers and community health agents. The investigator is responsible for overall activities and will regularly communicate the progress and possible problems met with his supervisor.

[PART ONE GENERAL 1](#__RefHeading___Toc231203859)

[1. Global tuberculosis control programme 1](#__RefHeading___Toc231203860)

[1.1. Global programme outline 1](#__RefHeading___Toc231203861)

[1.2. Global tuberculosis disease burden 2](#__RefHeading___Toc231203862)

[1.3 Tuberculosis control programme in Ethiopia 2](#__RefHeading___Toc231203863)

[1.4. Tuberculosis programme in southern region 2](#__RefHeading___Toc231203864)

[2. Statement of the problem 3](#__RefHeading___Toc231203865)

[3. Literature review 4](#__RefHeading___Toc231203866)

[4. Reason of the study 9](#__RefHeading___Toc231203867)

[5. Goal of the study 10](#__RefHeading___Toc231203868)

[5.1. General objective of the study 10](#__RefHeading___Toc231203869)

[5.2. Specific Objectives: 10](#__RefHeading___Toc231203870)

[6. Methods 13](#__RefHeading___Toc231203872)

[6.1. Study area and population 13](#__RefHeading___Toc231203873)

[6.3. Permission to continue 15](#__RefHeading___Toc231203874)

[6.4. Ethical clearance 15](#__RefHeading___Toc231203875)

[6.5. Data collection and handling 16](#__RefHeading___Toc231203876)

[6.6. Quality assurance 16](#__RefHeading___Toc231203877)

[6.7. Analysis plan 17](#__RefHeading___Toc231203878)

[7. Project management and work plan 17](#__RefHeading___Toc231203879)

[PART TWO INDIVIDUAL STUDIES 17](#__RefHeading___Toc231203880)

[STUDY ONE - TO EVALUATE THE DIAGNOSTIC VALUE AND APPLICABILITY OF COMMUNITY BASED TUBERCULOSIS SCREENING TOOL IN TUBERCULOSIS CASE DETECTION 17](#__RefHeading___Toc231203881)

[1. Objective 17](#__RefHeading___Toc231203882)

[2. Methods 17](#__RefHeading___Toc231203883)

[2.1. Study design 17](#__RefHeading___Toc231203884)

[2.2. Study variables 18](#__RefHeading___Toc231203885)

[2.3. Data collection techniques and tools 18](#__RefHeading___Toc231203886)

[2.4. Sample size calculation 19](#__RefHeading___Toc231203887)

[STUDY TWO - TO ESTIMATE THE TREATMENT OUTCOME OF TUBERCULOSIS PATIENTS SUPERVISED BY COMMUNITY HEALTH AGENTS/ TREATMENT SUPERVISORS 20](#__RefHeading___Toc231203888)

[1. Objective 20](#__RefHeading___Toc231203889)

[2. Methods 20](#__RefHeading___Toc231203890)

[2.1. Study design 20](#__RefHeading___Toc231203891)

[2.2. Study variables 22](#__RefHeading___Toc231203892)

[2.3. Data collection techniques and tools 22](#__RefHeading___Toc231203893)

[2.4. Sample size calculation 22](#__RefHeading___Toc231203894)

[STUDY THREE - TO FIND OUT THE COST PER TUBERCULOSIS PATIENT TREATED SUCCEFULLY 23](#__RefHeading___Toc231203895)

[1. Objective 23](#__RefHeading___Toc231203896)

[2. Methods 23](#__RefHeading___Toc231203897)

[2.1. Study design 23](#__RefHeading___Toc231203898)

[2.2. Study variables 24](#__RefHeading___Toc231203899)

[2.3. Data collection techniques and tools 24](#__RefHeading___Toc231203900)

[2.4. Sample size calculation 24](#__RefHeading___Toc231203901)

[STUDY FOUR - TO FIND OUT THE ACCEPTABILITY OF COMMUNITY BASED TB CARE 26](#__RefHeading___Toc231203902)

[1. Objective 26](#__RefHeading___Toc231203903)

[2. Methods 26](#__RefHeading___Toc231203904)

[2.1. Study design 26](#__RefHeading___Toc231203905)

[2.2. Study variables 26](#__RefHeading___Toc231203906)

[2.3. Data collection techniques and tools 26](#__RefHeading___Toc231203907)

[2.4. Sample size calculation 27](#__RefHeading___Toc231203908)

[STUDY FIVE - TO FIND OUT THE PROPORTION OF TUBERCULOSIS PATIENTS INFECTED WITH HIV 27](#__RefHeading___Toc231203909)

[1. Objective 27](#__RefHeading___Toc231203910)

[2. Methods 27](#__RefHeading___Toc231203911)

[2.1. Study design 27](#__RefHeading___Toc231203912)

[2.2. Study variables 28](#__RefHeading___Toc231203913)

[2.3. Data collection techniques and tools 28](#__RefHeading___Toc231203914)

[2.4. Sample size calculation 28](#__RefHeading___Toc231203915)

[References 29](#__RefHeading___Toc231203916)

[Annexes consent forms and questionnaires I](#__RefHeading___Toc231203917)

**PART ONE GENERAL**

**1. Global tuberculosis control programme**

**1.1. Global programme outline**

Mycobacterium tuberculosis has infected humans for thousands of years. The disease killed many patients[1]. However, even before introducing antituberculosis drugs, tuberculosis prevalence decreased in developed countries because of improved socio-economic conditions. Unfortunately, the situation worsened globally because of lack of enough control measures.

The World Health Organization (WHO) prioritizes tuberculosis control because of the high disease load, feasibility to carry out BCG vaccination and antituberculosis treatment. These raised the expectation for the coming up with effective strategy for tuberculosis prevention and control.

With the introduction Rifampicin, Directly observed short course therapy (DOTS) started as a strategy with five parts.[2] These are

1. Government commitment to ensure lasting and comprehensive tuberculosis control,
2. Case detection by sputum smear microscopy among self-reporting symptomatic patients,
3. Standardized short course chemotherapy using of six to eight months treatment regimens,
4. A regular and uninterrupted supply of all essential antituberculosis drugs,
5. A standardized recording and reporting[3]

Tuberculosis control programme started as a vertical programme. This approach was successful in developed countries where it was possible to undergo mass intervention. In developing countries, the approach worked well initially as the programme had its lowest coverage. However, with further programme expansion, it became difficult to oversee the programme from a centralized structure. This led to integrating the control programme into the general health service. Tuberculosis control programme integrated diagnostic service and treatment units followed by decentralization of executive roles.

Given the size of the tuberculosis problem, shortage of staff limits prevention and control of tuberculosis. Therefore, involvement of private-for-profit organizations, non-government organizations, community health workers and volunteers is needed.[2]

**1.2. Global tuberculosis disease burden**

In 2005, 15.4 million tuberculosis cases were reported globally. Among 8.8 million people were new tuberculosis cases of which 3.9 million were smear positive. The smear positive case detection rate is 42 %. The treatment success rate reached 82% and remained unchanged since then.

About 1.7 million people died of tuberculosis in the same year. Ninety eight percent of tuberculosis deaths occur in the developing countries, mainly affecting young adults. It is the leading cause of death among young women in Africa. If left unchecked, with in 20 years, tuberculosis will kill about 35 million people. [4, 5]

**1.3 Tuberculosis control programme in Ethiopia**

In Ethiopia, about 60 - 80 % of health problems are because of communicable diseases and malnutrition. Tuberculosis is among the leading causes of death and sickness in the country.[6] A well- organized tuberculosis control programme started in 1992 and the geographic coverage is 71%.[3]

Ethiopia ranks 7^th^ among high tuberculosis burden countries in the world. Ninety five percent of health institutions give DOTS service for population. Unfortunately, 40% of population do not have access to health service. The annual incidence and prevalence of all forms of tuberculosis is 356 and 533 per 10^5^ populations respectively. The case notification rate for all forms tuberculosis is 166 per 10^5^ populations. [4] The annual incidence of smear positive tuberculosis is 155 cases 10^5^ populations. The case notification of smear positive tuberculosis cases is 56 10^5^ populations. The case detection rate of all forms and smear positive cases is 47 % and 36 % respectively. The proportion of patients who are cured and completed treatment (treatment success rate) is about 76 %. [4]

**1.4. Tuberculosis programme in southern region**

Southern Nations, Nationalities and peoples Regional State (SNNPRS) is one of the Federal

States of Ethiopia. The region has a population of about 14 million. Ninety-three percent of the population live in rural areas. The health service coverage and user rate is about 50 % and 32 %, respectively.

In 1995, DOTS started as a pilot project in three zones and four health institutions of the southern region of Ethiopia. Now, all hospitals and health centres provide DOTS to tuberculosis patients. As a result, number of tuberculosis cases has increased. However, the case detection rate was not in proportion to the programme coverage.[7]

Low coverage of the health service, low use rate and poverty compromised access to tuberculosis care. As a result, the case detection rate is below the global target. Thus, we need to find better ways of addressing the tuberculosis problem.[7]

**2. Statement of the problem**

Ten years have passed since tuberculosis prevention and control programme was started in the southern region of Ethiopia. Experiences from the programme implementation for a decade showed the following findings. In the first five years, the programme was vertical and centralized with slow expansion. The case detection rate has increased compared with the existed tuberculosis programme coverage. However, many patients failed to adhere to treatment and follow up to give sputum specimen for laboratory examination. The next five years there was better programme coverage and the cure and treatment completion rates has improved. However, the case detection remained low.

Currently the case detection rate of smear positive cases is only 39 %. The challenges were shortage of health workers, low health service coverage, low health service user rate, high disease burden and socioeconomic barriers. Despite the challenges, tuberculosis prevention and control programme demands uninterrupted supplies, regular supervision, and strict adherence to DOTS.

The low health service coverage compromises tuberculosis control. This gives opportunity for disease transmission and increases disease burden in the community. In addition, increasing the health service coverage and training health workers to fill the gap in short period seems a remote possibility. On the other side, improving health seeking behaviour and changing the low socio economic status would need longer period.

Therefore, tuberculosis control programme should find alternative ways that increase access to diagnostic and treatment service to tuberculosis patients. This will increase tuberculosis case detection and treatment. It may also decrease patient delays and lead to early initiation of treatment. This will decrease the risk of tuberculosis transmission in the community. This study aims at improving community based tuberculosis care in Ethiopia.

**3. Literature review**

Over the last two decades, because of the overlapping HIV AIDS pandemic, the number of active tuberculosis has increased in sub-Saharan Africa. This led to increased workload on the health services because of the dual epidemics. This needs major adjustment in tuberculosis control programme so patients could get better care.

As a result, tuberculosis control programmes opted for different tuberculosis treatment supervision approaches. One of these was self-supervised therapy. In this alternative, patients receive drugs with out supervisory visit. Compared with facility-based tuberculosis care, self-supervised therapy improved treatment outcome. However, it needed intensive health education and regular patient supervision to increase adherence to treatment. In other studies, the treatment success rate was lower than institution based tuberculosis care. They self-supervised therapy in situations where direct supervision was not practical or was refused by the patients.[8]

Some studies adjusted the approach to improve patient adherence to treatment, namely modified DOTS. In this approach, they tried to reduce the number of tuberculosis patient visits to tuberculosis clinic. However, to compensate for reduced patient visits, health workers regularly visited patients and delivered health education to the patients. Sometimes, tuberculosis patients received incentives and enablers as meals and refunding transport costs so that patients will adhere to treatment. [9] This also leads to increased programme cost and workload on health workers.

In addition, regular home visits by health workers encouraged tuberculosis patients adherence to treatment. This showed improved patient treatment success rate and decreased defaulter rates. However, maintaining regular health workers visits remained as a challenge. The study recommended motivation of health workers to attain better patient care. [10] However, this is difficult to achieve, as there is shortage of health workers. The problem is that without direct supervision of patients, the possibility of completion of treatment is unpredictable and low.

A consensus statement released by group of practitioners underlined that patient centred treatment strategy as a benchmark for tuberculosis control. The main reason is that it is more acceptable to the patient in his or her way of life than clinic or clinician centred treatment. In addition, it was more successful regardless of the country, community or number of supervision carried out. Therefore, this needs organizing tuberculosis control programme in such a way that it will improve access, increase adherence to treatment and lessen socioeconomic burden. [9] And it should be within the existing community supported by a strong social and political network.[11]

Experience of community based tuberculosis care showed an improved tuberculosis treatment outcome in different settings. In Tanzania, community based tuberculosis treatment using guardian, as a treatment supervisor was as effective as facility-based tuberculosis treatment in urban settings. It showed that community based tuberculosis care is complementary to the conventional approach.[12] However, it was not possible to identify the margin between guardian and self-supervised treatment when it comes to direct treatment observation.

In Swaziland, they compared tuberculosis treatment supervision by community health workers and family members. They found that treatment supervision by family members was equally effective compared with supervision by community health workers. However, they recommended that selection of treatment supervisors should consider patient preference and access.[11, 13] As seen from above, the studies showed that community-based tuberculosis treatment is as effective as and could complement facility-based tuberculosis treatment. However, the variation of tuberculosis treatment supervisors from place to place remained a challenge to recommend the best supervisor under different settings.

On the other hand, improving tuberculosis case detection is one of the targets and main challenges of tuberculosis control. To solve this, investigators used different approaches to estimate the size of the disease in the community. Tuberculosis prevalence surveys conducted used methods like home visits, small x-rays and mobile diagnostic services. However, the number of tuberculosis cases detected was low in countries with low tuberculosis prevalence and the cost per case identified was too high to put into practice in poor countries.

WHO, as well as national tuberculosis control programme of Ethiopia, recommends ways to improve the case detection under the provision of self-reporting of patients to health facilities. These are public health education on early self- reporting to examination, training of health workers and making diagnostic facilities accessible to the patients.[3]

Improving the knowledge of general health workers will increase the index of suspicion of health workers to detect tuberculosis cases. In addition, it will improve the quality of information delivered to tuberculosis patients during treatment initiation and follow-up. In a study conducted in Tigray the relationship between the knowledge of community health agents and the health institutions mainly depends on the quality of care that the health institution is delivering. [14] This in turn might improve the health seeking behaviour of tuberculosis patients and hence case finding.

Other investigators suggested symptom based tuberculosis screening in clinical and community setting when resources are limited [15-17]. Therefore, case detection requires simple and applicable community based tuberculosis case finding tools to detect cases as early as possible to deal with delayed presentation of patients to health institutions.

Late presentation of tuberculosis patients to medical care is still a major barrier to tuberculosis case finding. Study from southern Ethiopia showed the median delay in presentation to be about 4 months. Of these 75 % of patients had duration of illness more than two months.[18] In urban settings, the median patient delay was two months in addition to the health service delay of about one week. The delay is longer for patient living far away from health facilities and with low knowledge about TB. They recommended further decentralization of the service [19, 20]and introduction of feasible strategies that improve patient referral as early as possible. [21] Moreover, the association of poverty and stigma attached to tuberculosis played big role in increasing patient delay. So carrying out activities that focus on making tuberculosis care accessible to the poor and the rural community is important.[22]

Other reasons for delay are the inability of health workers to detect tuberculosis patients in the clinic. Therefore, understanding the main presenting symptoms of tuberculosis by health workers is important to increase tuberculosis case detection. Some studies identified the main symptoms described by tuberculosis patients. Specifically cough, night sweats, weight loss and tiredness are among the symptoms with high predictive value.[23, 24] A study conducted in Ethiopia described the main symptoms of tuberculosis patients and found that about 95 % had cough as a presenting symptom after two weeks.[25]

Health workers should have high index of suspicion of tuberculosis to increase case detection. This demands simple symptom score to improve diagnosis. This may reduce the duration of health service delay. This has been a practice for childhood tuberculosis case finding though it was not conclusive to date.[26] A study carried out in Ethiopia used tuberculosis symptoms and x-ray results to identify its diagnostic value. They found sensitivity and specificity of above 90%. However, about 40 % patients were below the cut-off point.[27] In addition, the cost and low access of radiological services in the country remains a practical problem to tuberculosis patients. Therefore, simple and applicable tuberculosis scoring system would play an important role in case finding by health and community health workers.

In addition, the nature of mycobacterium gives an opportunity to improve case finding. Mycobacterium tuberculosis remains viable for longer period when stored at a room temperature. A Study from Malawi showed that it could stay viable for about four weeks.[28] The fact that the bacilli could stay viable for longer time under room temperature in a humid set up could be an opportunity to tuberculosis control programme. Therefore, community health workers could collect and transport sputum specimen over short period for laboratory examination for tuberculosis bacilli.

Hence, the access to diagnostic health facilities might improve as well as the frequency of visits and cost incurred by the patients might decrease. One of the challenges to this approach is the non-adherence to the national diagnostic guidelines. [29] However, strengthening quality control system could minimize the problem. In addition, a study group in southern Ethiopia recommended regular on job training to laboratory technicians and regular supervision to improve the performance of laboratory technicians in peripheral diagnostic institutions. [30]

To be more pragmatic, implementation of tuberculosis control programme should consider the available inputs. In resource-constrained settings, programme managers opt for an intervention with low cost for improved programme indicators. Economic evaluations of tuberculosis control programme showed that treating tuberculosis is a cost-effective intervention. They further recommended additional effort to increase tuberculosis case finding and improve accessibility of service to the community in line with millennium development goals. [31]

Cost of community based tuberculosis care was determined in different countries. In Bangladesh, they compared the cost incurred by non-governmental organizations (NGOs) with that of the government for tuberculosis control programme. The NGOs used community health workers in tuberculosis case finding and patient follow-up. The cure rate was nearly equal in the two settings. However, the cost per tuberculosis patient cured by NGO was two thirds of government tuberculosis programme. The investigators recommended that collaboration with NGOs could improve tuberculosis care. However, the cost of sputum microscopy was higher than in the government programme.[32] They could also reduce the cost by strictly adhering to WHO recommendations for case finding.

In a study conducted in Uganda, they evaluated conventional hospital-based and community-based tuberculosis treatment for all tuberculosis patients. They admitted all smear positive tuberculosis patients in hospital for two months. Community based approach reduced the total cost per patient treated successfully by 43 % and increased success rate of smear positive tuberculosis patients by 19 %. The main drop in cost was because of the decreased duration of hospital admission from two months to nineteen days on average.[33] This mainly focused on hospital settings. Therefore, the cost of treating tuberculosis patients at health centre level needs further analysis.

An individual randomized controlled trial study conducted in Tanzania found that community-based tuberculosis treatment reduced cost per tuberculosis patient successfully treated by 35 % with similar treatment outcomes. The main cost drop was because of fewer visits to tuberculosis clinic. They recommended applicability of community based tuberculosis care in resource limited settings.[34] Before applying to a wider set-up, they should further evaluate the applicability at community level.

The World Bank reported that tuberculosis control programme as a cost-effective health intervention. [35] Different studies showed that community-based tuberculosis care is better than the existing conventional tuberculosis care. It also showed that it is less costly per patient treated and as effective or more compared to all alternative approaches [9, 36]. Therefore, this could be a better and feasible option in resource-constrained countries with high tuberculosis burden and overstretched health care.

In summary, community-based approach compliments the conventional facility based treatment. It minimizes cost per patients treated successfully and reduces the work burden on health workers. It is equally acceptable as health facility based treatment[37], improves patient adherence to treatment, and follow-up. Moreover, by incorporating simple symptom score, community health workers could assist in tuberculosis case finding and sputum collection and transport. Therefore, tuberculosis control programme should explore ways of improving community-based tuberculosis care in different settings.

**4. Reason of the study**

Over the last two decades, the load of tuberculosis has increased in sub-Saharan Africa. The main reasons are human immunodeficiency virus infection and poverty. This led to an increased burden on the health services because of the associated morbidity and mortality. This led to health service exhaustion and forces patients to attend health institutions often.

The conventional passive case finding mostly serves those who have the access, knowledge, better health seeking behaviour and better socioeconomic status to go to health institutions. As tuberculosis patients are mostly from low socio-economic class, this will further lead to patient and health service delay and increase tuberculosis transmission in the community.

In addition, getting access to health institutions for diagnosis and treatment is expensive and difficult for tuberculosis patients. Therefore, adherence and completion of full course anti-tuberculosis drugs remains a challenge to tuberculosis patients. This demands alternative approaches to improve access to diagnosis and treatment.

In Ethiopia, improving health service coverage and increasing the number of health workers to fill the gap seems a distant possibility. However, carrying out acceptable tuberculosis control in the community is important to complement the existing overstretched health service to improve health seeking behaviour and increase tuberculosis case finding. Therefore, using community-based approach to fill the gap in the tuberculosis control programme remains an unexplored alternative.

So far, there has not been any study in southern Ethiopia to improve community based tuberculosis care. This study aims at improving community based tuberculosis care in southern Ethiopia.

**5**. **Goal of the study**

The goal of this research is to improve the tuberculosis control programme of Ethiopia

**5.1. General objective of the study**

To improve interventions to carry out community based tuberculosis control programme in southern Ethiopia.

**5.2. Specific Objectives:**

1. To evaluate the diagnostic value and applicability of community based tuberculosis screening tool in tuberculosis case detection,
2. To estimate the treatment outcome of tuberculosis patients supervised by community health agents or tuberculosis treatment supervisors
3. To find out the costs per tuberculosis patient treated successfully
4. To find out the acceptability of community based directly observed short course therapy
5. To find out the proportion of tuberculosis patients infected by human immunodeficiency virus

**Table** - **Summary of the individual studies**

| **Paper** | **Objectives** | **Study design** | **Study population** | **Duration** |
| --- | --- | --- | --- | --- |
| I. | To evaluate the diagnostic value and applicability of community based tuberculosis screening tool in tuberculosis case detection | Cross-sectional study | TB suspects older than 15 years old | Sep 2006/ Mar 2008 |
| II. | To estimate the treatment outcome of tuberculosis patients supervised by community health agents or tuberculosis treatment supervisors | Randomized control community trial | TB patients | Sep 2006/ Mar 2008 |
| III. | To find out the costs per tuberculosis patient treated successfully | Randomized control community trial | Health workers and TB patients | Sep 2006/ Mar 2008 |
| IV. | To find out the acceptability of community based directly observed short course therapy | Cross-sectional study | Health workers and TB patients | Mar 2007/ Mar 2008 |
| V. | To find out the proportion of tuberculosis patients infected human immunodeficiency virus | Cross-sectional study/survey | TB patients older than 15 years old | May 2006/ Oct 2006 |

**The research project in relation to the public health model of tuberculosis control**

Non infected

TB infected

Disease development

Recognition of symptoms

Health care seeking

Getting diagnosis

Treatment compliance

Treatment outcome

Study III - Cost per TB

patient treated successfully

Study I - diagnostic value and applicability of TB symptom score

Study II - estimate treatment outcome of TB patients

Study IV - acceptability of community based

tuberculosis care

**Post TB social consequences**

Study V - estimate the proportion of tuberculosis patients infected with HIV

**6.** **Methods**

**6.1. Study area and population**

Southern region is the third large stand populous regions in Ethiopia. It is located in the southwest part of the country with a population of 14.08 million. It has thirteen zones and eight special districts. The health service coverage is 50 %. However, the user rate is only 32 %. Tuberculosis diagnostic facilities are only located in health centres and less accessible to the patients.

Sidama zone is in southern region of Ethiopia. It has 10 districts and 2 city administrations. It is one of the most densely populated areas of the region with a population of about 2.8 million. It has one hospital, 20 health centres, 26 health stations and 85 health posts. The health service coverage is 55%.

In 1996, the DOTS started in Sidama. Currently all health facilities except all health posts deliver DOTS. The zone reports many tuberculosis cases. In addition, there are many community health workers in the zone. Some of these are community based reproductive health agents, traditional birth attendants, community health promoters, health extension workers and others. Practically so far, they have not participated in tuberculosis control programme.

**6.2.** **Study design**

**Study - I**

This is a cross-sectional study to evaluate the diagnostic value and applicability of community based tuberculosis-screening tool in tuberculosis case finding. The investigator will select matched clusters and randomly allocate to intervention and non-intervention clusters. Community health agents from the intervention clusters will organize community mobilization sessions monthly and register patients with cough of two weeks or more. Then they will run the diagnostic tool. Patients with symptoms suggestive of pulmonary tuberculosis will give sputum specimen according to the recommendation of national tuberculosis guideline. The community health agent will keep the sputum specimen in a box prepared for this purpose and transport the sputum specimen, the same day, to diagnostic health institution for laboratory examination. Laboratory technicians will stain the sputum specimen using by Ziehl-Neelsen technique on the same day. Then they will do direct microscopic examination. They will make the results ready for the next day. In addition, they will send sputum specimen for fluorescent microscopy, bleach digestion technique and culture. Patients in the non-intervention clusters will continue the conventional self-reporting and investigation in diagnostic health facilities.

**Study - II**

This is a randomized controlled community trial to estimate the treatment outcome of tuberculosis patients treated by community based directly observed short course therapy. The investigator will select clusters and match for tuberculosis treatment outcome. Then will randomly allocate them to intervention and control clusters. Health workers and community health agents in the intervention clusters will receive training on community-based tuberculosis care and its implementation. They will also discuss with diagnosed tuberculosis patients about the disease and selection of treatment supervisor. Tuberculosis patients will select tuberculosis treatment supervisor from their area and discuss with health worker about their role and responsibility. Once agreed up on, tuberculosis patients will start supervised short course therapy in their community under tuberculosis treatment supervisor. The study will follow the national tuberculosis programme guideline for patient diagnosis, treatment, follow-up, recording and reporting. Patients in the non-intervention clusters will continue conventional health-facility based directly observed short course therapy.

**Study - III**

This is a randomized controlled community trial to find out the cost per tuberculosis patient treated successfully. The study follows societal perspective of cost analysis. The main cost items related to the costs of tuberculosis prevention and control programme will be collected as for diagnosis, follow up and supervision including patient and community health workers time and costs. Data collectors will also get records from district finance offices. The data collectors will calculate cost of time based on monthly salary of health workers and tuberculosis patients if they are in the productive age groups. In addition, the cost of the person accompanying the patient will be included. The outcome indicator for the study is the cost per patient treated successfully.

**Study - IV**

This is a cross-sectional study to find the acceptability of community based tuberculosis care by health workers, community members, and tuberculosis patients. Health workers, tuberculosis patients and community members will participate in the study districts. The data collectors will randomly select tuberculosis patients, health workers, health extension workers, tuberculosis treatment supervisors and community health agents who are directly involved in tuberculosis patient care. From the community previously treated tuberculosis patients and community members living closer to tuberculosis patients, next fifth household heads will be taken. Trained data collectors will administer a pretested questionnaire about the acceptability of community-based TB care.

**Study - V**

This is a cross-sectional study or survey to find the proportion of tuberculosis patients infected with human immunodeficiency virus. The investigator will select ten sites from the region as part of regional surveillance of HIV infection among tuberculosis patients. The study will include all tuberculosis patients diagnosed over six months period. Trained laboratory technicians and health workers from tuberculosis clinic /data collectors/ will administer pr-tested questionnaire and record socio-demographic variables as age, sex, residence, classification and category of tuberculosis. Tuberculosis patients will give serum for HIV testing. The laboratory technicians will label and store the blood specimen as per the recommendation for HIV testing. They will transport the specimen to regional reference laboratory under cold chain. Trained laboratory technologists in the regional reference laboratory will do HIV testing under national testing algorithm. The results will be anonymous and unlinked.

## 6.3. Permission to continue

The investigator will discuss with the national tuberculosis prevention and control programme about the procedure and all the supplies needed as drugs, reagents, sputum cups, and formats. In addition, the investigator will discuss with authorities and tuberculosis programme coordinators at all levels the regional health bureau, zonal, district, health institutions and the community leaders the aim and the implementation of the intervention study.

**6.4. Ethical clearance**

The investigator will get ethical clearance from University of Bergen and Southern Nations, Nationalities and Peoples Regional Health Bureau Ethical Review Committee.

**6.5. Data collection and handling**

In collaboration with programme coordinators, the investigator will train health workers and community health agents according to the training guideline. Except for those specifically prepared for the study, will use formats used by the national tuberculosis control programme formats. Training of data collectors will focus on community-based tuberculosis care activities, individual studies, data collection techniques and administering a questionnaire. The training will have practical pretesting session. Data collectors will receive field guides and checklists prepared as a reference for the study period.

Health workers and community health agents will give questionnaires completed to the investigator or supervisors. The supervisors will check the completeness and accuracy of the questionnaires. The investigator will file and keep the filled questionnaires.

**6.6. Quality assurance**

Community health agents will be trained about community based tuberculosis care, identifying tuberculosis suspects, sputum collection and handling, recording and reporting, selection of tuberculosis treatment supervisor, and drug and supply management. The data collectors will receive training materials, checklists and field guides for field activities.

The research project and the national tuberculosis control programme will work together during the study period to help easy take over of the activity after the end of the study. Supervision schedule will be set in partnership with the follow-up plan of the health care system of the region except for specific supervision schedules for the intervention.

Tuberculosis programme coordinators will supervise tuberculosis treatment supervisors during follow-up. The programme coordinator and the investigator will supervise the health institutions. In addition, the investigator, zonal and regional tuberculosis programme coordinators, will supervise data collectors and treatment supervisors.

Field supervisors will contact tuberculosis patients, treatment supervisors, community members and health workers. In addition, they will cross-check five per cent of the data in the community with tuberculosis unit register in health institutions and woreda tuberculosis registers. The programme coordinator alone, or with the principal investigator will supervise the activities on weekly basis to provide supplies, do rapid assessment of the general activities and give timely solution to the problems met. Reference laboratory technologists will take five percent of tuberculosis slides done in health facilities for quality control. In addition, two independent technologists in the reference laboratory will check HIV test results.

**6.7. Analysis plan**

The investigator will check the data, sort and review it manually for errors and inconstancies. Then will use SPSS 13.1 for Windows for analysis.

# 7. Project management and work plan

The principal investigator, in teamwork with tuberculosis programme coordinators, local community leaders and community health workers, will oversee the tuberculosis control in the study areas. He will also take care of the administrative issues, overall help of the field activities and regular checking of the project progress. The principal investigator will keep proper records of the research and regularly communicate the progress and possible problems met with his supervisor.

**PART TWO INDIVIDUAL STUDIES**

**STUDY ONE - TO EVALUATE THE DIAGNOSTIC VALUE AND APPLICABILITY OF COMMUNITY BASED TUBERCULOSIS SCREENING TOOL IN TUBERCULOSIS CASE DETECTION**

**1. Objective**

- to evaluate the diagnostic value and applicability of community based tuberculosis screening tool in tuberculosis case detection

**2. Methods**

**2.1. Study design**

This is a cross-sectional study to evaluate the diagnostic value and applicability of community based tuberculosis-screening tool in tuberculosis case finding. The investigator will divide study sites into different clusters and match them based on the previous tuberculosis case finding and treatment outcome. He will pair the matched clusters. Then he will randomly allocate the paired clusters into intervention and control groups. This study will be carried out in intervention clusters parallel with the second study on treatment outcome. The study will include 15 years or more tuberculosis suspects who experienced productive cough of two or more weeks.

Trained community health workers will organize sessions of community mobilization on monthly basis. They will teach about tuberculosis its prevention and control. Community health workers will interview community members who have the symptoms suggestive of tuberculosis. Then they will run the diagnostic tool. All tuberculosis suspects who meet the criteria will give sputum specimen as per the national guideline recommendation. Community health workers will collect the sputum specimen label it and keep it in a box prepared to transport the specimen to diagnostic health facilities. Laboratory technicians will do direct sputum microscopic examination for acid-fast bacilli using Ziehl-Neelsen staining technique. The laboratory technicians will also send sputum specimen to Yirgalem hospital for culture, bleach digestion and fluorescent microscopy.

**2.2.** **Study variables**

The study variables are the socio-demographic characteristics as age, sex, education, marital status, tuberculosis symptoms, the duration and history related to tuberculosis.

**2.3. Data collection techniques and tools**

As part of tuberculosis prevention and control strategy, community health agents will be trained how to identify tuberculosis suspects from the community. They will also receive practical training about using the diagnostic tool, sputum collection, and coding, labelling, and handling technique. Tuberculosis programme will supply sputum cups and sputum collection boxes for community health agents. They will set up referral system among the health institutions, the laboratory technicians and community health agents. In addition, will set dates for sputum transporting, smear result, treatment and follow up of patients.

Community health workers will organize sessions of community mobilization every month. They will run the diagnostic tool. All tuberculosis suspects who meet the criteria will give sputum specimen for laboratory examination. Tuberculosis suspects will give sputum specimen as per the national guideline. They will give the first sputum specimen at spot after running the diagnostic tool. They will go home with the second labelled sputum cup and come next day with morning sputum. They will give the third sputum specimen when coming with the second next day. Community health workers will collect all sputum specimens, fill laboratory request formats, label sputum cups as per the national tuberculosis programme recommendation and keep it in a box prepared for sputum store in the community. Community health workers will transport the sputum specimen and deliver it to the laboratory technicians in the diagnostic health institution.

The laboratory technicians will do three direct sputum microscopy examinations by using Ziehl-Neelsen technique on the same day when they received sputum specimen. They will make the results ready for the next day. In addition, laboratory technicians will send the sputum specimens to Yirgalem hospital for culture, bleach digestion technique and fluorescent microscopy. We will use Lowenstein-Jenson medium under standard culture requirement to grow mycobacterium tuberculosis. Two independent trained laboratory technicians will do the microscopic examinations. The laboratory technicians will send a signed result to third independent record keeper. The investigator will check the results and report those patients who turned out to be smear positive for tuberculosis. Health workers will start them on antituberculosis treatment as per the national tuberculosis manual.

Laboratory technicians in the diagnostic health institutions health centres and hospital will keep all the slides examined for mycobacterium tuberculosis. The regional reference laboratory will do external quality control check for five per cent of the laboratory results for all specimens.

**2.4. Sample size calculation**

The proportion of pulmonary tuberculosis cases detected by Sidama tuberculosis control programme is about 20%.

n = $\frac{z_{\left[1-\alpha\right]}^{2}*pq}{\left[d\right]^{2}}$

Where: n = the sample size

p = the proportion of pulmonary tuberculosis patients detected

q = 1- p

z_α =_ the percentage level at 95% confidence interval (1.96)

d = the margin of error = 5%

Based on this

n = $\frac{\left[1.96\right]^{2}*0.2*\left(1-0.2\right)}{\left(0.05\right)^{2}}$ = 246

**STUDY TWO - TO ESTIMATE THE TREATMENT OUTCOME OF TUBERCULOSIS PATIENTS SUPERVISED BY COMMUNITY HEALTH AGENTS/ TREATMENT SUPERVISORS**

**1. Objective**

- to estimate the treatment outcome of tuberculosis patients supervised by community health agents/ treatment supervisors

**2. Methods**

**2.1. Study design**

This is a randomized controlled community trial to estimate the tuberculosis treatment outcome through community based tuberculosis care. The investigator will select clusters from study districts. Based on the population, health service coverage and mainly on tuberculosis treatment outcome the investigator will match the clusters. He will match clusters and randomly assign them into intervention and non-intervention groups.

**Intervention sites**

The investigator, in collaboration with tuberculosis programme coordinators, will give training to health workers and community health agents about community based tuberculosis care and their role and responsibilities. The training will focus on tuberculosis case finding, case holding, drug management, recording and reporting as per the national guideline for community based tuberculosis prevention and control. The trainees will receive course materials and field guides to the trainees.

All diagnosed tuberculosis patients in the selected clusters will be included in the study. Health workers will inform tuberculosis patients about the disease and the selection criteria of treatment supervisors. Patients will select and come to health workers for discussion about treatment supervision. Once agreed upon, the health worker will discuss with treatment supervisor about community based tuberculosis care, their role and responsibility during the course of treatment.

Selection criteria for treatment supervisor selection is as follows: a volunteer leaving in the community who can read and write, can comprehend health education about tuberculosis prevention and control, has time to give daily dose as per the recommendation, can follow the patient through out the treatment duration and assist the patient during the follow up and drug collection. It is necessary that the treatment supervisors should have a known address so that supervisors from health facilities, Woreda, or investigator can contact during supervisory field visits in the course of patient management. Parents or guardians will supervise treatment of their children in paediatric age group less than 13 years if they can assume the role of treatment supervisor as per the selection criteria. If not, they will identify treatment supervisor.

Tuberculosis patients, who completed intensive phase in health facilities and patients on re-treatment regimen will not participate in the study. Treatment supervisors, who could not supervise at least the first two months of treatment, will be excluded.

Health workers will register tuberculosis patients on programme and research formats prepared for community based tuberculosis care. Tuberculosis treatment supervisors will also register the patients on the daily treatment follow up format. Health workers will record tuberculosis case finding and treatment outcome of the intervention sites. They will submit research formats to the investigator and will keep copies of their health institutions.

The regional tuberculosis control programme will supply all the needed drug and supplies to the respective districts and facilities via existing health care system. The scheme of supervision will have the following pattern: tuberculosis treatment supervisors will supervise tuberculosis patients. Health extension workers will supervise tuberculosis treatment supervisors. Health workers will supervise both activities. The investigator and coordinators at all levels - regional, zonal, and district will supervise health facilities, health extension workers, tuberculosis treatment supervisors and the patients as the regional supervision scheme.

**Non-intervention sites**

Tuberculosis patients in the non-intervention district will follow the existing facility based tuberculosis case finding and treatment. They will receive supplies and programme managers will carry out supervision according to the existing supervision system.

**Reporting**

District programme managers will report tuberculosis control activities according to the existing reporting system of the region as it carried out on quarterly bases. Reports include cases detected, treated, sputum follow up, drug, supervision laboratory supplies, and request for next quarter. In addition, they will submit research formats to the investigator. They will keep copies of their office.

**2.2. Study variables**

The variables included are socio-demographic, knowledge and attitude towards tuberculosis and the patients, and factors contributing to the willingness to supervise treatment. Community health workers will ask for any antituberculosis drug side effect.

**2.3. Data collection techniques and tools**

Health workers will register all the required information about tuberculosis patients as per the national recommendation and the study. At the end, health workers will hand over all records related to the study to the investigator. They will also report their activities to the health institutions, district, zone and regional level. Data collectors will collect data from the reports and formats prepared for community-based tuberculosis care.

**2.4. Sample size calculation**

The treatment success rate of Sidama zone is 70 %. With the intervention, we assume effect size of 21 % so that to increase the treatment success to 85%, WHO target.

n = [(z_1_ + z_2_)*2*P$\left(1-P\right)$]/$\left(p_{1}-p_{2}\right)$

n = the sample size from each group z_1_ =level of significance of 95 %( 1.96)

z_2_ =power of the study of 90 %( 1.28) p_1_ =proportion of treatment success in control group (70%)

p_2_ =proportion of treatment success in intervention group (85%)

P= the average proportion of treatment success in the control and intervention groups (77.5%)

n =$\frac{\left[\left(z_{1}+z_{2}\right)^{2}*2P\left(1-P\right)\right]}{\left(p_{1}-p_{2}\right)^{2}}$ = $\frac{\left[\left(1.96+1.28\right)^{2}*2*0.775*\left(1-0.775\right)\right]}{\left(0.85-0.70\right)^{2}}$ = 163

IF = 1 + (m-1)*ICC

IF - inflation factor or design effect m - cluster size /15 TB patients

[Based on tuberculosis prevalence of 533 per 100,000, taking case detection rate of 47% as reported by WHO 2005, we expect 15 tuberculosis cases per village of 6000 population.]

ICC - intracluster correlation coefficient assuming typical ICC of 0.04

IF = 1 + (15 - 1) * 0.04 = 1.56 Effective sample size = IF * calculated size = 1.56 * 163 = 255

To calculate number of clusters per group

Total tuberculosis patients divided by cluster size 255 /15 = 17 clusters per group

k - Coefficient of variation = standard deviation of proportions/mean of proportions p_1_ = 70% p_2_= 85% P = 77.5% SD =0.106 k =0.12

c = 2 + $\left(z_{1}+z_{2}\right)^{2}$ $\left[\frac{2P\left(1-P\right)}{n}+k^{2}\left(p_{^{1}}^{2}+p^{2}\right)\right]$/$\left(p_{1}-p_{2}\right)^{2}$

c = 2 + $\left(1.96+1.28\right)^{2}$ $\left[\frac{2*0.775\left(1-0.775\right)}{30}+0.12^{2}\left(0.7^{2}+0.85^{2}\right)\right]$/$\left(0.85-0.7\right)^{2}$ = 14 clusters

The case detection of all forms of tuberculosis is 47 % of the annual incidence of 356 cases per 10^5^ populations. Assuming that the case detection of all forms of tuberculosis will increase to 70%, we expect case detection of 250 cases per 10^5^ populations. Therefore, to detect 30 cases per cluster we need about 12,000 people. A Kebele /villages have a population of about 6000, in densely populated areas as in Sidama. So a cluster will have two kebeles.) With this case detection, the study will take one year for case detection and additional 6- 8 months to record treatment outcome.

**STUDY THREE - TO FIND OUT THE COST PER TUBERCULOSIS PATIENT TREATED SUCCEFULLY**

**1. Objective**

- To find out the costs per tuberculosis patient treated successfully

**2. Methods**

**2.1. Study design**

This is a randomized controlled community trial to find out the costs per tuberculosis patient treated successfully in the intervention and non-intervention districts. Cost related to tuberculosis control programme in the study sites will be determined according to the actual expenses and time cost. Programme coordinators, health workers, community health agents, tuberculosis treatment supervisors, tuberculosis patients and relatives accompanying them to health facilities in the study sites will be included in the study.

The cost analysis will follow societal perspective to analyse the cost per patient. The data collectors will obtain financial reports from districts and health institutions finance sections to collect expenditures for tuberculosis control programme, health workers and community health agents’ time and costs associated to tuberculosis prevention and control activities. In addition, they will collect cost of the patient and the relative who accompanied them during their visit to health institutions.

The investigator will use cost per tuberculosis patient successfully treated as an outcome measure. Future cost and productivity gains will not be included in the study. Standard cost estimation of all expenses and time of the patient, treatment supervisor and health workers will be done. Data collectors will use a structured pre-tested questionnaire for data collection.

**2.2. Study variables**

The study variables are the socio-demographic characteristics, time, cost incurred during tuberculosis case detection, follow up and treatment. In addition cost related to supervision and monitoring and evaluation of tuberculosis control programme in the study sites will be included.

**2.3. Data collection techniques and tools**

Trained data collectors will administer a pre-tested structured questionnaire. Study variables include socio-demographic variables of tuberculosis patients, financial expenses and time. When the tuberculosis patient is less than 18 years, the data collectors will interview the parents or the guardians accompanying them to health institution. The data collectors will review financial reports of health institutions and the district.

**2.4. Sample size calculation**

Community based tuberculosis care reduced 30% of patient cost in health facility to 13 % in community-based tuberculosis treatment in a study conducted in Tanzania.[34] Based on this

n = [(z_1_ + z_2_)*2*P$\left(1-P\right)$]/$\left(p_{1}-p_{2}\right)$

n = the sample size from each district z_1_ =level of significance of 95 %( 1.96)

z_2_ =power of the study of 90 %( 1.28) p_1_ = patient cost in facility based tuberculosis treatment (30%)

p_2_ = patient cost in community-based tuberculosis treatment (13%)

P= the average proportion of treatment success in the control and intervention groups (21.5%)

n =$\frac{\left[\left(z_{1}+z_{2}\right)^{2}*2P\left(1-P\right)\right]}{\left(p_{1}-p_{2}\right)^{2}}$ = $\frac{\left[\left(1.96+1.28\right)^{2}*2*0.215*\left(1-0.215\right)\right]}{\left(0.13-0.30\right)^{2}}$ = 123

IF = 1 + (m-1)*ICC

IF - inflation factor or design effect m - number of cluster size 15 assuming equal cost per cluster

ICC - intracluster correlation coefficient assuming typical ICC of 0.04

IF = 1 + (m - 1)*ICC = 1 + (15 - 1) * 0.04 = 1.56

Effective sample size = IF * calculated size = 1.56* 123 = 192

In addition, the cost related to tuberculosis treatment supervisors, community health agents, tuberculosis programme coordinators in the district and health institution will be included.

**STUDY FOUR - TO FIND OUT THE ACCEPTABILITY OF COMMUNITY BASED TB CARE**

**1. Objective**

- to assess acceptability of community based tuberculosis care

**2. Methods**

**2.1. Study design**

This is a cross-sectional study to find out the acceptability of community based tuberculosis care. The study will include all health workers (health professionals, health extension workers and community health agents), community members, and tuberculosis patients who completed their treatment (ex tuberculosis patients) in the study sites. Based on the sample size, study subjects will be randomly selected from all intervention clusters. Community members next to treated tuberculosis patients will be included in the study. Next fifth household on the right side of treated tuberculosis patients’ house will be included. Health workers working in tuberculosis clinic, health extension workers and tuberculosis treatment supervisors will be included in the study.

**2.2. Study variables**

The study variables are the socio-demographic characteristics and factors related to acceptability indicators why they accept, how they want it to be, who should deliver the service and others.

**2.3. Data collection techniques and tools**

Data collectors will find the names of ex tuberculosis patients from tuberculosis unit registers and list them out according to the study clusters. Based on the number and sample size they will randomly select ex tuberculosis patients from all intervention cluster to participate in the study.

During data collection if they found that the ex tuberculosis patient has died or moved to other community they will replace her/him by next ex tuberculosis patient on the list. If they come across ex tuberculosis patient less than 18 years old, they will interview head or guardian of the household.

In addition to find out the acceptability by the community, data collectors will interview household next to ex tuberculosis patients household if they know about tuberculosis treatment. If they do not know will proceed on the next right side household and interview guardian head of the household.

A standardized pretested questionnaire including socio-demographic variables and factors affecting acceptability will be included in the questionnaire. Programme coordinators and the investigator will give training to health workers about administering the questionnaire. Trained data collectors will administer the interview preferably by those who can speak the local language.

**2.4. Sample size calculation**

In a study conducted in the Tigray, northern part of Ethiopia, it showed that 64.5 % community health workers accepted community based tuberculosis care. [38]

n = $\frac{z_{\left[1-\alpha\right]}^{2}*pq}{\left[d\right]^{2}}$

Where: n = the sample size

p = the proportion of tuberculosis patients who accepted community-based tuberculosis care

q = 1- p z_α =_ the percentage level at 95% confidence interval (1.96)

d = the margin of error = 5% Based on this

n = $\frac{\left[1.96\right]^{2}*0.645*\left(1-0.645\right)}{\left(0.05\right)^{2}}$ = 351

**STUDY FIVE - TO FIND OUT THE PROPORTION OF TUBERCULOSIS PATIENTS INFECTED WITH HIV**

**1. Objective**

- to find out the proportion of tuberculosis patients infected with HIV

**2. Methods**

**2.1. Study design**

This is a cross-sectional study/survey to find out the proportion of tuberculosis patients infected with HIV in the region. As part of regional tuberculosis and HIV infection, the investigator will select nine sites from the region. Selection criteria of the sites are that they should have laboratory technician, well performing DOTS programme, laboratory reagents, functional x-ray machine, functional refrigerator to store blood sample and be accessible for supervision. All tuberculosis 15 years old or more patients on treatment will be included in the study.

**2.2. Study variables**

The study variables are the socio-demographic characteristics age, sex, address, type and category of tuberculosis.

**2.3. Data collection techniques and tools**

All patients who are diagnosed to have TB will be asked to submit 5ml blood sample to the laboratory for routine lab investigations. The health worker in tuberculosis clinic will assign a unique study number to each patient on the data collection format. The laboratory technician will draw blood specimen by sterile technique using disposable needle, syringe and gloves. He or she will label the blood specimen with the same unique study number assigned by health worker in Tb clinic without any other identifier. They will keep the samples in a refrigerator with optimum temperature. The laboratory technician or health worker from TB clinic will transport the specimen in cold chain and deliver to the regional laboratory.

Laboratory technologists will conduct anonymous HIV testing according to the national HIV testing algorithm. They will register the results on the data collection format and submit to principal investigator.

**2.4. Sample size calculation**

n = $\frac{z_{\left[1-\alpha\right]}^{2}*pq}{\left[d\right]^{2}}$

Where: n = the sample size

p = the proportion of pulmonary tuberculosis patients infected with HIV q = 1- p

z_α =_ the percentage level at 95% confidence interval (1.96) d = the margin of error = 2.5%

Study from the region showed that the prevalence of TB HIV co-infection rate of about 20%.[39]

Based on this

n = $\frac{\left[1.96\right]^{2}*0.2*\left(1-0.2\right)}{\left(0.025\right)^{2}}$ = 984

**References**

1. Warring, F.C., Jr., *A brief history of tuberculosis.* Conn Med, 1981. **45**(3): p. 177-85.

2. M C Raviglione, A.P., *Evolution of WHO policies for tuberculosis control, 1948–2001.* THE LANCET, 2002. **359**: p. 775–80.

3. *Tuberculosis and Leprosy Prevention and Control Manual, Ministry of Health, Ethiopia.* 2002. **2nd Edition**.

4. *Global Tuberculosis Control Surveillance, Planning, Financing*. 2005, World Health Organization.

5. *WHO fact book, tuberculosis the global burden*. 2005.

6. *Health and health related indicators*, p.a.p.t.o.M.o.H.o. Ethiopia, Editor. 2003/04.

7. *Tuberculosis Leprosy and Blindness prevention and control program, Annual report*. 2004, Southern Nations, Nationalities and Peoples Regional Health Bureau, Awassa.

8. R Prasad, D.M.R., Surya Kant and A Jain, *A comparison of unsupervised treatment along with intensive health education and directly obsereved treatment in pulmonary tuberculosis.* Ind J Tub, 2001. **48**(21).

9. Chaulk, C.P. and V.A. Kazandjian, *Directly observed therapy for treatment completion of pulmonary tuberculosis: Consensus Statement of the Public Health Tuberculosis Guidelines Panel.* Jama, 1998. **279**(12): p. 943-8.

10. Jin, B.W., et al., *The impact of intensified supervisory activities on tuberculosis treatment.* Tuber Lung Dis, 1993. **74**(4): p. 267-72.

11. *Community TB care: Practice and Policy*. Vol. WHO/CDS/TB/2003.312. 2003.

12. Wandwalo, E., et al., *Effectiveness of community-based directly observed treatment for tuberculosis in an urban setting in Tanzania: a randomised controlled trial.* Int J Tuberc Lung Dis, 2004. **8**(10): p. 1248-54.

13. Wright, J., et al., *Direct observation of treatment for tuberculosis: a randomized controlled trial of community health workers versus family members.* Trop Med Int Health, 2004. **9**(5): p. 559-65.

14. Mengiste M Mesfin, T.W.T., Isreal G Tareke and Madeley RJ Richard., *Community health workers: their knowledge on pulmonary tuberculosis and willingness to be treatment supervisorsin Tigray, northern Ethiopia.* The Ethiopian Journal of Health Development, 2005. **Volume 19,**(Special issue, 2005,): p. 1-34.

15. Santha, T., et al., *Are community surveys to detect tuberculosis in high prevalence areas useful? Results of a comparative study from Tiruvallur District, South India.* Int J Tuberc Lung Dis, 2003. **7**(3): p. 258-65.

16. Golub, J.E., et al., *Active case finding of tuberculosis: historical perspective and future prospects.* Int J Tuberc Lung Dis, 2005. **9**(11): p. 1183-203.

17. A. K. Chakraborty, R.C., M.S. Krishina Murthy, A. N. Shashidhara, V. V. Krishina Murthy and K. Chaudhuri, *Prevalence of pulmonary tuberculosis in a peri-urban community of Bangalore under various methods of population screening.* Ind J Tub, 1994. **41**: p. 17.

18. Madebo, T. and B. Lindtjorn, *Delay in Treatment of Pulmonary Tuberculosis: An Analysis of Symptom Duration Among Ethiopian Patients.* MedGenMed, 1999: p. E6.

19. Demissie, M., B. Lindtjorn, and Y. Berhane, *Patient and health service delay in the diagnosis of pulmonary tuberculosis in Ethiopia.* BMC Public Health, 2002. **2**: p. 23.

20. Yimer, S., G. Bjune, and G. Alene, *Diagnostic and treatment delay among pulmonary tuberculosis patients in Ethiopia: a cross sectional study.* BMC Infect Dis, 2005. **5**(1): p. 112.

21. Mengiste M Mesfin, T.W.T., Isreal G Tareke, Yohannes T Kifle, Witten H Karen, and Madeley J Richard, *Delays and care seeking behavior among tuberculosis patients in Tigray of northern Ethiopia.* Ethiopian Journal of Health Development, 2005. **19**(special): p. 7 - 12.

22. Cambanis, A., et al., *Rural poverty and delayed presentation to tuberculosis services in Ethiopia.* Trop Med Int Health, 2005. **10**(4): p. 330-5.

23. G. Rathman, J.S., P. C. Hill, J. F. Murray, R. Adegbola, T. Corrah, C. Lienhardt, and K.P.W.J. McAdam, *Clinical and radiological presentation of 340 adults with smear-positive tuberculosis in The Gambia.* INT J TUBERC LUNG DIS, 2003. **7**(10): p. 942–947.

24. El-Sony, A.I., et al., *Symptoms in patients attending services for diagnosis of pulmonary tuberculosis in Sudan.* Int J Tuberc Lung Dis, 2003. **7**(6): p. 550-5.

25. Teklu, B., *Symptoms of pulmonary tuberculosis in consecutive smear-positive cases treated in Ethiopia.* Tuber Lung Dis, 1993. **74**(2): p. 126-8.

26. Mehnaz, A. and F. Arif, *Applicability of scoring chart in the early detection of tuberculosis in children.* J Coll Physicians Surg Pak, 2005. **15**(9): p. 543-6.

27. Tessema, T.A., et al., *An evaluation of the diagnostic value of clinical and radiological manifestations in patients attending the addis ababa tuberculosis centre.* Scand J Infect Dis, 2001. **33**(5): p. 355-61.

28. Banda, H.T., et al., *Viability of stored sputum specimens for smear microscopy and culture.* Int J Tuberc Lung Dis, 2000. **4**(3): p. 272-4.

29. Mengiste M Mesfin, T.W.T.a.M.J.R., *The quality of tuberculosis diagnosis in districts of Tigray region of northern Ethiopia.* The Ethiopian Journal of Health Development, 2005. **Volume 19**(Special issue, 2005,): p. 1-34.

30. Estifanos Biru Sharegie, M.A.Y.B.L., *Quality control of sputum microscopic examinations for acid fast bacilli in southern Ethiopia.* Ethiopian Journal of Health Development, 2005. **19**(2): p. 104 - 108.

31. Baltussen, R., K. Floyd, and C. Dye, *Cost effectiveness analysis of strategies for tuberculosis control in developing countries.* Bmj, 2005. **331**(7529): p. 1364.

32. Islam, M.A., et al., *Cost-effectiveness of community health workers in tuberculosis control in Bangladesh.* Bull World Health Organ, 2002. **80**(6): p. 445-50.

33. Okello, D., et al., *Cost and cost-effectiveness of community-based care for tuberculosis patients in rural Uganda.* Int J Tuberc Lung Dis, 2003. **7**(9 Suppl 1): p. S72-9.

34. Wandwalo, E., B. Robberstad, and O. Morkve, *Cost and cost-effectiveness of community based and health facility based directly observed treatment of tuberculosis in Dar es Salaam, Tanzania.* Cost Eff Resour Alloc, 2005. **3**: p. 6.

35. Musgrove, P., *Investing in health: the 1993 World Development Report of the World Bank.* Bull Pan Am Health Organ, 1993. **27**(3): p. 284-6.

36. Floyd, K., D. Wilkinson, and C. Gilks, *Comparison of cost effectiveness of directly observed treatment (DOT) and conventionally delivered treatment for tuberculosis: experience from rural South Africa.* Bmj, 1997. **315**(7120): p. 1407-11.

37. Wandwalo, E., et al., *Acceptability of community and health facility-based directly observed treatment of tuberculosis in Tanzanian urban setting.* Health Policy, 2005.

38. Mengiste M Mesfin, T.W.T., Israel G Tareke and Madeley RJ Richard., *Community health workers: their knowledge on pulmonary*

*tuberculosis and willingness to be treatment supervisors in*

*Tigray, northern Ethiopia.* 2005. **19**(Special issue): p. 1 - 34.

39. Yassin, M.A., et al., *HIV and tuberculosis coinfection in the southern region of Ethiopia: a prospective epidemiological study.* Scand J Infect Dis, 2004. **36**(9): p. 670-3.

**Annexes consent forms and questionnaires**

**Community consent form Improving community based tuberculosis care in southern Ethiopia**

Request of participation

Ethiopia is among high tuberculosis burden countries in the world. As a result, our region shares similar problem. The conventional method of treating tuberculosis mainly focuses on health institution based directly observed treatment. Tuberculosis patients travel to health facilities on daily bases to get the treatment. This costs them extra time and money they expend for diagnosis and treatment.

Our study aims at improving community-based tuberculosis care by making the service available in the community whereby community health workers and tuberculosis treatment supporters treat the patients. The advantage of the intervention is that patients will not be visiting health facilities daily. This will in turn decrease the related expenses and improve adherence.

Tuberculosis patients in the community will have equal access to the diagnostic and treatment facilities as other patients in the region. There is no obligation and related punishment in case you do not like to take part in the study. Your participation in the study is fully based on voluntary decision. You have the right to participate and withdraw from the study. Every patient in the community will get the detail information about the intervention and the decision will be left for them as to participate or not in the study.

In case of inconveniences or for more information, Dr. Daniel Gemechu will be available during supervision and you could contact him through health workers in the community and use the following address.

Box 303 Awassa,

Tele. 00 251 46 2202847

In addition, you have the right to ask tuberculosis treatment supporters, health professionals in the

health institutions and district programme managers.

**Individual consent form: improving community based tuberculosis care in southern Ethiopia**

**Purpose of the study**: you are invited to join the study because you have tuberculosis. All study participants will receive the standard treatment for tuberculosis according to the guidelines of Ministry of health. The purpose of this study is to see whether the patients who get tuberculosis treatment in their own community have better treatment outcome and minimize cost compared to other tuberculosis patients who travel to health facilities on daily basis to collect and swallow their drugs. Your village is selected to participate in the study and we have discussed with the leaders of the village and district

**What participation involves**: in order to participate in the study we will ask you to identify someone who will assist you in treatment supervision during treatment period. You will get your daily treatment from the person you selected. She/he will be responsible and the contact person between you and the health facility. You will inform her/him in case you have any problem regarding the treatment or the follow up. Tuberculosis programme managers from different places will also supervise you.

**Confidentiality**: all information collected from you will be kept confidential and will not be disclosed to others. It will only be used for the purpose of the study.

**Risks**: we do not expect any risk in participating in the study since the drugs you are taking are safe. In case you develop side effects from the drugs inform to your treatment supervisor and we will make sure that you get the appropriate treatment if indicated.

**Rights to withdraw and alternatives**: Taking part in the study is completely your choice. If you decide to stop participating in the study, you will receive all the treatments available for tuberculosis patient in the nearby health facility.

**Benefits**: the anticipated benefits of participating in the study are that you do not have to travel to health facilities on daily basis and this will make you pay less for travel expenses and save your time. The results of this study will be of great benefit to other tuberculosis patient in the region.

**Who to contact** if you have any question you can contact your treatment supporter, health facility tuberculosis programme coordinators, village leaders and district managers. If you still have problem you could contact, Dr. Daniel Gemechu, the investigator.

Do you have any question?

I, have read and clearly understood the contents of this form. My questions have been answered. I agree to participate in the study.

Signature of the participant Signature of the research assistant

Date Date

**TUBERCULOSIS SYMPTOM BASED SCREENING TOOL**

**Questionnaire no.________ Name of interviewer ______________________Date__________**

**1. Socio-demographic variables**

1.1 Name of suspect _______________________________ 1.2. Age_____1.3. Sex ___________

1.4. Cluster __________ Kebele ______________Residence urban _____ rural ______________

1.5. Marital status Single___________ Married_____________ Divorced________________

Widowed_______ other (specify) _________________________________

1.6. Educational status No schooling ______ Grade ______ other (specify) ________________

1.7. Occupation of suspect Farmer _________ student ________ merchant ________________

Housewife _________government employee________________________

daily labourer__________ others (specify) _________________________

**2. Tuberculosis symptoms and history**

|  | Tuberculosis symptoms and history | No | Yes | Duration in weeks |
| --- | --- | --- | --- | --- |
| 2.1. | Did you experience cough for two or more weeks? |  |  |  |
| 2.2 | Is the cough productive of sputum? |  |  |  |
| 2.3. | Does sputum contain blood? |  |  |  |
| 2.4. | Did you have fever? |  |  |  |
| 2.5. | Did you have loss of appetite? |  |  |  |
| 2.6. | Did you loss weight? |  |  |  |
| 2.7. | Did you have chest pain? |  |  |  |
| 2.8. | Did you have history of tuberculosis treatment? |  |  |  |
| 2.9. | Did you have closer contact with known tuberculosis patient? |  |  |  |

**COMMUNITY HEALTH WORKERS FIELD LAB REQUEST FORM**

**Unique number ________**

**Socio-demographic variables**

1. Name of TB suspect _________________________________ 2. Age________3. Sex _______

4. Cluster _______________ Kebele ______________________ Date ___________________

**Sputum specimen for microscopic examination**

**Sputum specimen sputum cup number slide number sputum result**

1^st^ spot ___________ _________ ________

2^nd^ morning ___________ ________ ________

3^rd^ spot ___________ ________ ________

**Name of community health agent Name of laboratory technician**

**Signature Signature**

**LAB RESULT OF SPUTUM EXAMINATION IN YAH**

**Unique number ________**

**Socio-demographic variables**

1. Name of TB suspect _____________________________________ 2. Age_____3. Sex ______

4. Cluster _______________ Kebele ______________________ Date ___________________

**Sputum specimen for microscopic examination and culture**

**Sputum specimen sputum cup number FM result Bleach result culture result**

1^st^ spot ______________ _________ ________ ___________

2^nd^ morning _____________ _________ ________ ___________

3^rd^ spot ________________ _________ ________ ___________

**Name of lab. technician - FM Name of lab. technician - bleach Name of lab. technician culture**

**Signature Signature Signature**

**Questionnaire: Community based tuberculosis treatment outcome**

**Identification**

1. District TB number ________________Unit TB number ____________

2. District_______Cluster number______Kebele _________Date_______

**Socio-demographic variables**

3. Age _______ 4. Sex: ____ 5. Residence urban ______ rural _______

6. Marital status Single _ Married _ Divorced _ Widowed _other (specify)_

7. Educational level No schooling __ Grade ____other (specify) ________

8. Occupation Farmer ________ student ________ merchant ________

Housewife _______government employee______________

Daily labourer_______ others (specify) ________________

**Patient follow up and treatment outcome**

1. Disease classification PTB+ve ______ PTB -ve ______ EPTB ________

2. Patient category New____ Relapse____ Failure_____ Defaulter______

Transfer in____ other (specify) _________________

3. Sputum smear results of PTB +ve patients

2^nd^ month __________ 5^th^ month _________ 7^th^ month _______

4. Treatment outcome cured ___ treatment completed ___ died ______

Defaulted ____ failure ____ transfer out _______

Other (specify) ___________________________

**COMMUNITY BASED TUBERCULOSIS CARE – TB PATIENT FORM**

Questionnaire no.________ Name of interviewer __________ Date___________

**Socio-demographic variables**

1. Name of TB patient___________________ 2. Age________3. Sex _____

4. Cluster ________________ Kebele ___________ 5. Religion ______

6. Marital status Single___ Married___ Divorced___ Widowed____ other___

7. Educational status No schooling ____ Grade ____ other ________

8. Occupation of suspect Farmer ____ student ___ merchant ___others _

**Cost items for diagnosis for the patient**

1. number of travel days ____ travel expense per day ____travel hours__
2. expenses for food ________ drink _______ others with expense _____

**Cost items for diagnosis for the relative if accompanied**

1. number of travel days _____ travel expense per day ___travel hours__
2. number of hours spent in the health institution per day ____________
3. expenses for food ______ drink _________ others with expense _____

**Cost items for treatment and follow up of the patient**

1. number of travel days _____ travel expense per day ___travel hours__
2. number of hours spent in the health institution per day ____________
3. expenses for food _______ drink ________ others with expense _____

**COMMUNITY BASED TUBERCULOSIS CARE – LAB TECHNICIAN FORM**

**Questionnaire no.__________ Name of interviewer ___________ Date___________**

**Socio-demographic variables**

1. Name of health worker _________________ 2. Age________3. Sex ____

4. Health facility ______________ profession __________ 5. Religion __

6. Monthly income _______________

7. Marital status Single___ Married___ Divorced___ Widowed____ other___

**Cost items for laboratory sputum microscopy**

1. number of hours taken for explaining about sputum specimen collection __
2. number of hours taken for sputum staining, microscopy and reporting____

**Cost items for laboratory for follow up sputum**

1. number of hours taken for explaining about sputum specimen collection
2. number of hours taken for sputum staining, microscopy and reporting__

**COMMUNITY BASED TUBERCULOSIS CARE – TB OPD FORM**

**Questionnaire no.__________ Name of interviewer ___________ Date___________**

**Socio-demographic variables**

1. Name of health worker __________________ 2. Age________3. Sex __

4. Health facility ____________ profession ___________ 5. Religion __

6. Monthly income _______________

7. Marital status Single___ Married___ Divorced___ Widowed____ other___

**Cost items for daily dose of intensive phase treatment**

1. number of hours taken for explaining about the disease to the patient _
2. number of hours taken for daily dose supervision and registration _____

**Cost items for continuation phase treatment**

1. number of hours taken for explaining about the disease process ______
2. number of hours taken for drug administration and registration _______

**COMMUNITY BASED TUBERCULOSIS CARE – TTS FORM**

**Questionnaire no.________ Name of interviewer __________ Date___________**

**Socio-demographic variables**

1. Name of TTS________________________ 2. Age________3. Sex ______

4. Cluster ________________ Kebele ___________ 5. Religion _______

6. Marital status Single___ Married___ Divorced___ Widowed____ other____

7. Educational status No schooling ____ Grade ____ other ________

8. Occupation of suspect Farmer ___ student ___ merchant ____others __

**Cost items for treatment supervision**

1. number of hours spent per for treatment supervision ______________
2. total number of days tuberculosis patient is supervised ____________
3. number of travel hours for drug collection _______________________
4. number of days traveled for drug collection ______________________
5. travel expenses per day _____________________________________
6. related expenses for food ___________ drink __________ others ___

**COMMUNITY BASED TUBERCULOSIS CARE – CHW FORM**

**Questionnaire no.________ Name of interviewer __________ Date__________**

**Socio-demographic variables**

1. Name of CHW_______________________ 2. Age________3. Sex ______

4. Cluster ________________ Kebele ___________ 5. Religion _______

6. Marital status Single___ Married___ Divorced___ Widowed____ other____

7. Educational status No schooling ____ Grade ____ other ________

8. Occupation of suspect Farmer ___ student ___ merchant ____others __

9. Monthly income __________

**Cost items for sputum collection**

1. number of hours spent per community education _________________
2. total number of days community mobilization conducted____________
3. total number of hours spent for sputum collection ________________
4. total number sputum collected per session ______________________
5. number of hours spent on sputum transportation _________________
6. number of days sputum is transported to health facilities ___________
7. number of hours spent for sputum result collection _______________

**Cost items for treatment**

17. Number of hours spent about sputum result and TTS selection ________

18. Number of hours spent in discussion with TTS _____________________

19. Number of hours spent on drug collection from health facilities ________

20. Number of days traveled for drug collection _______________________

21. Expenses for food ________ travel _________ others _______________

22. Number of hours spent on supervision ___________________________

23. Number of days supervision is conducted _________________________

**COMMUNITY BASED TUBERCULOSIS CARE – DISTRICT FORM**

**Questionnaire no.________ Name of interviewer __________ Date___________**

**Resources for tuberculosis control**

1. Health manpower for tuberculosis control

Profession____________________ monthly salary________ __________

2. Motor bike________________ number ___________

3. Budget allocated for tuberculosis control programme _________________

**Cost items for supervision**

4. Number of supervisory visits conducted __________________________

5. Number of hours spent per supervision __________________________

6. Number of health facilities supervised per supervision ________________

7. Number of community health workers supervised per supervision ______

8. Number of kilometers traveled per supervision _____________________

9. Total expenditure for fuel for supervision of TB programme____________

* other costs will be obtained from the finance report

**Serosurvey among tuberculosis patients in southern region**

Study site ______________ Date ___________

Patient code no _______________ Age __________ Sex __________

Address Urban ___________ Rural _______________

Diseases category a) New b) Relapse c) treatment after default

Disease classification a) smear positive b) smear Negative c) EPTB

Name of responsible health worker ________________

Signature ____________

**Acceptability of community tuberculosis care - TB patients**

Identification

1. Name______________________

2. District_________Cluster ______Kebele`___________

Socio-demographic variables

3. Age _________ 4. Sex: _____

4. Marital status Single __ Married __ Divorced __ Widowed ___Other___

5. Religion Protestant ___ Muslim ___ Orthodox ___ Other ____

6. Educational level No schooling ___ Grade ________

7. Occupation Farmer__Govt employee__Student__Housewife__other___

Acceptability questions

1. Do you accept tuberculosis treatment in the community Yes __ No _

2. If yes, why?

a) Time

b) Cost

c) Travel

d) Environment

e) Type of supervisor

f) Understanding each other

g) Flexible time place

h) Better care and adequate time for discussion

i) Trust each other

j) Decrease stigma and discrimination

k) Could do it with out worrying other members

l) others specify

3. If No, Why?

a) Professional incompetence

b) Poor drug storing

c) Stigma

d) specify

**CURRICULUM VITAE**

**1. Personal information**

Full Name: Daniel Gemechu Datiko Date of Birth: February 20/1973

Sex: Male Place of Birth: Gidole, Ethiopia

Marital Status: Married Nationality: Ethiopian

Religion: Protestant

**2. Educational Background**

- Doctor of Medicine (MD) from Addis Ababa University (1993 - 1999)

**3. Work Experience**

- July 2003 - July 2005: Regional TB Leprosy and Blindness programme coordinator
- January - July 2003 : Yirgalem Hospital Sidama Zone, Assistant Medical Director
- April 2001 - July 2003 : Yirgalem Hospital Sidama Zone, General medical Practitioner
- October 1999 - April 2001 : Darara Health Centre, General medical Practitioner

**5. Language ability**:

1. Amharic - Reading , Writing and Speaking
2. English - Reading , Writing and Speaking

**6. Professional Membership**

- Member of Ethiopia Public Health Association (EPHA)

**7. Professional Interest**

- Tuberculosis prevention and control
- Community based interventions

**8. References**

- Dr Shiferaw Tekle MariamHabtamu, Head of regional Health Bureau, Awassa

[shiferaw_t@hotmail.com](../shiferaw_t@hotmail.com), [t_kmariam@yahoo.com](../t_kmariam@yahoo.com), Tele: +251 46 2203263

**9. Address**

University of Bergen, Centre for International Health, Armeur Hanson Building,

Box 5023, Bergen, Norway

E-mail: [Gemechu.Daniel@student.uib.no](../Gemechu.Daniel@student.uib.no%20)

Po Box: 303

Awassa, Ethiopia

Tele: Res: +251 46 2202847

E-mail: [danieljohn42@yahoo.com](../danieljohn42@yahoo.com%20)

I hereby notify that, to the best of my knowledge and ability the information given above is genuine and true.

Date Signature _____________________ ________________________
